# Evaluating the Impact of *Lactobacillus acidophilus* on *Fusarium* Mycotoxins in Raw Vegan Pumpkin–Sunflower Seed Flour Blends

**DOI:** 10.3390/foods14173077

**Published:** 2025-09-01

**Authors:** Iveta Brožková, Marek Pernica, Sylvie Běláková, Markéta Vydržalová, Petra Moťková, Ivana Stará, Lenka Husáková, Libor Červenka

**Affiliations:** 1 Department of Biological and Biochemical Sciences, Faculty of Chemical Technology, University of Pardubice, Studentská 573, 532 10 Pardubice, Czech Republic; iveta.brozkova@upce.cz (I.B.); marketa.vydrzalova@upce.cz (M.V.); petra.motkova@upce.cz (P.M.); ivana.stara@student.upce.cz (I.S.); 2 Research Institute of Brewing and Malting, Malting Institute, Mostecká 7, 614 00 Brno, Czech Republic; pernica@beerresearch.cz (M.P.); belakova@beerresearch.cz (S.B.); 3 Department of Analytical Chemistry, Faculty of Chemical Technology, University of Pardubice, Studentská 573, 532 10 Pardubice, Czech Republic; lenka.husakova@upce.cz

**Keywords:** contamination, vitarianism, drying, competition, principal component analysis

## Abstract

A blend with pumpkin and sunflower seed flours was prepared and dried at 41.5 °C for 5 h to create a minimally heat-treated blend for a raw food diet. The blend was inoculated with *Lactobacillus acidophilus* and *Fusarium langsethiae* to assess the effect of *L. acidophilus* on *Fusarium* growth and mycotoxin production. Drying did not affect the content of naturally occurring microorganisms but significantly reduced water activity (*p* < 0.05) and increased total phenolic content in samples with external microorganisms. *Lactobacilli* content remained unchanged after drying (4.8 log CFU/g), while *F. langsethiae* increased by 1.5 log CFU/g. Principal component analysis showed PC1 explained 95.1% of total variance, driven by *Fusarium* mycotoxin production. A significant difference in total mycotoxin was found between samples with *F. langsethiae* alone and those with both *F. langsethiae* and *L. acidophilus* (*p* < 0.05). Lactic acid bacteria could reduce fusarium mycotoxin risk in raw food diet mixtures.

## 1. Introduction

Interest in alternative diets has risen with improvements in living standards. Individuals adopt these patterns for varied reasons, including ethical and religious beliefs, environmental and animal-welfare concerns, and health considerations. Despite their diversity, these approaches share an emphasis on holistic well-being and balance with nature [1,2]. The raw food diet—also termed raw foodism (rawism) or vitarianism—emphasizes consuming foods not heated above ~42–45 °C [3]. Such diets may include fruits, vegetables, seeds and nuts, eggs, meat, fish, seaweed, honey, milk, and other dairy products. They can also encompass items processed without high heat, for example, fermented foods (sauerkraut, kimchi), cheese, yoghurt or kefir, as well as dehydrated or frozen foods and cold-pressed oils. Conversely, treatments such as homogenisation and pasteurization are typically excluded, and the use of pesticides, fertilizers, and food additives is generally avoided [3,4].

Consuming minimally heat-treated (MHT) food may retain more heat-labile nutrients. Such diets are typically rich in fibre, minerals, antioxidants, and vitamins [5]. They may also be associated with lower total fat intake and, consequently, lower LDL cholesterol levels [6]. Vitarianism has been reported to be associated with reduced risk of cardiovascular diseases and certain cancers [4].

MHT foods generally contain fewer added ingredients, undergo less processing, and may be perceived as easier to digest. Individuals who adopt such diets may experience weight loss, increased energy, and clearer skin [4]. However, general conclusions about the overall benefits of raw food diet cannot be drawn. A review of 18 clinical trials concluded the diets comprising more than 90% raw foods are not recommended for long-term adherence because of micronutrient deficiencies [7]. Another concern is the increased risk of contamination with pathogenic bacteria with common infections including listeriosis, salmonellosis, and yersiniosis [8].

*Fusarium* mycotoxins, most prominently the trichothecenes (T-2 toxin, HT-2 toxin), zearalenone, and fumonisins, pose a major concern in agriculture and food safety owing to their toxicity in humans and animals. Produced by *Fusarium* spp., these compounds frequently contaminate cereal crops such as wheat, barley, and maize. They exhibit considerable stability under diverse environmental conditions, including certain food-processing operations. Consumption of contaminated foods may result in immunosuppressive, carcinogenic, and mutagenic effects [9]. In Europe, regulations have been set to limit the acceptable levels of these toxins in food and feed; nevertheless, contamination continues to cause significant economic losses [10]. Recently, Pernica et al. reported the presence of *Fusarium* species and high levels of T-2 toxin and its metabolites in all 15 spring barley samples harvested in the Czech Republic [11], underscoring the need to reduce *Fusarium* mycotoxin burdens along the food chain.

Mycotoxin production can be countered by lactic acid bacteria (LAB), commonly present in fermented foods. Research indicates that effects are strain-specific, with certain *Lactobacillus* spp., and related LAB such as *Lactococcus lactis* subsp. lactis is able to mitigate or prevent mycotoxin production by *Fusarium* spp. Inhibition is attributed in part to organic acids’ production (e.g., lactic, acetic), which lowers extracellular pH; undissociated acids can enter fungal cells and dissociate, collapsing the H+ gradient and impairing growth [12]. Additional contributions may include nutrient competition and related resource limitations that constrain mycotoxin biosynthesis [13,14,15]. Moreover, recent reports propose that the predominant mechanism of mycotoxin removal by LAB is physical adsorption to cell-wall components rather than biodegradation [16,17].

Demand for minimally processed, plant-based foods is increasing, largely motivated by ‘clean-label’ preferences [18]. Analyses of Google search behaviour across 23 countries indicate that veganism and vegetarianism are among the most frequently queried diet types [19]. In this context, our work is directly relevant: we evaluate a consumer-available, minimally heat-treated seed-and-fruit blend representative of raw vegan preparations, a matrix in which elevated water activity and modest temperatures can pose a risk of *Fusarium* growth and mycotoxin production. The aim of this study is to evaluate the effect of *Lactobacillus acidophilus* on *Fusarium* mycotoxins in pumpkin–sunflower seed flour blends (specific food for raw food diet).

## 2. Materials and Methods

### 2.1. Media

Representative samples of raw ingredients (10 or 2 g) were homogenized with 90 or 98 mL of peptone salt solution (or peptone water for dates), constituting a 10^−1^ dilution. Serial decimal dilutions (10^−2^, 10^−3^, 10^−4^) were prepared in a sterile peptone-salt solution. Total microbial count (TBC) was determined on plate count agar (HiMedia, Laboratories, Maharashtra, India). Dilutions 10^−3^ and 10^−4^ were plated to enumerate mesophilic aerobic and facultative anaerobic microorganisms, using the pour-plate method (1 mL inoculum) and spread-plate method (0.1 mL inoculum). Plates were incubated at 30 °C for 24–48 h.

Total coliform bacteria count (TCBC) was enumerated on violet red bile lactose agar (HiMedia, Laboratories, Maharashtra, India). Dilutions of 10^−1^ and 10^−2^ were plated by the pour-plate method (1 mL inoculum). Plates were incubated at 30 °C for 24–48 h, and colonies with characteristic red-purple appearance were counted.

Total yeasts and moulds count (TYMC) were enumerated on dichloran rose bengal medium (HiMedia Laboratories, Maharashtra, India) formulated for samples with water activity > 0.95. Aliquots (0.1 mL) of the 10^−2^ and 10^−3^ dilutions were spread onto the agar surface and incubated at 25 °C for 5 days.

Osmophilic yeasts and xerophilic moulds count (OXC) were enumerated on a dichloran-glycerol (DG18) medium (HiMedia Laboratories, Maharashtra, India). Inoculation was performed by the pour-plate method using 0.1 mL of the 10^−1^ and 10^−2^ dilutions. Plates were incubated at 25 °C for 30 days.

Presumptive *Bacillus cereus* count(BCC) was enumerated on mannitol-egg yolk-polymyxin (MYP) agar (HiMedia Laboratories, Maharashtra, India). Aliquots (0.1 mL) of the 10^−1^ and 10^−2^ dilutions were spread onto the agar surface and plates were incubated at 37 °C for 24–48 h.

### 2.2. Microbiological Analysis of Ingredients for MHT Blend Production

The MHT blend, a mixture of various raw ingredients, was prepared following a recipe found online for vitarianism (RAWMANIA.cz). The first step involved microbiological testing of each component listed in Table S1.

Two appropriate dilutions were plated in duplicate onto Petri dishes (90 mm in diameter). The total number of microorganisms *N* (CFU per g) was calculated using equation:

*N* = ∑*c*/(*V*·(*n*_1_ + 0.1·*n*_2_)·*d*), (1)

where ∑*c* = the sum of the number of colonies of two consecutive dilutions; *V* = inoculated volume of bacterial solution (mL); *n*_1_ = the number of Petri dishes used for the calculation from the lower dilution; *n*_2_ = the number of Petri dishes used for the calculation from the higher dilution; *d* = the dilution factor corresponding to the lower dilution [20]. The results were expressed as log CFU per gram of the sample.

After incubation, counts were calculated according to Equation (1) and expressed as colony-forming units per gram (CFU/g). Fungi were identified by macro- and microscopic morphology. Bacteria and yeasts were identified based on colony appearance on the respective media, Gram staining, and biochemical tests as specified in the relevant standards. Isolates of the genus *Bacillus* were identified by MALDI-TOF/MS (in collaboration with MeDiLa, spol. s r. o., Pardubice, Czech Republic).

Microbiological analysis of the blend and MHT blend was performed as follows. Portions (10 g) of each sample were homogenized with 90 mL peptone-salt solution using a Masticator Basic (IUL, Barcelona, Spain). The 10^−1^ and 10^−2^ dilutions were plated onto the relevant solid media as described in Section 2.1. *Lactobacillus* counts were additionally determined on *Lactobacillus* MRS agar by spreading 0.1 mL of the appropriate dilution with a sterile L-shaped spreader. Plates were incubated at 37 °C for 3 days under microaerophilic conditions, and counts of *Lactobacillus* spp. were calculated (1).

### 2.3. Preparation of Inoculum

*Lactobacillus acidophilus* CCM 4833 (Czech Collection of Microorganisms, Masaryk University, Brno, Czech Republic) was stored freeze-dried at −80 °C. The bacteria were activated by transferring onto MRS agar (HiMedia Laboratories, Maharashtra, India) and incubating at 37 °C for 48 h under microaerophilic condition. This strain was chosen as a food-relevant lactic acid bacterium with recognized safety and acidification capacity, not previously tested for modulating *Fusarium* mycotoxins in semi-solid matrices. *Fusarium langsethiae* (barley isolate) was included as a representative trichothecenes producer isolated in Czechia [11]. The fungal isolate was activated on malt extract agar (MEA; HiMedia Laboratories, Maharashtra, India) plates at 25 °C for 7 days. A colony was then transferred to MEA slants and incubated at 25 °C for a further 7 days. Spores were harvested by vortexing the slants with 5.0 mL sterile saline containing 0.1% of Tween 80. The suspension was homogenized with glass beads, and spore concentration was determined using a cell-counting chamber.

### 2.4. The Preparation of MHT Blend and Inoculation of Lactobacillus acidophilus and Fusarium langsethiae

The MHT blend was prepared following the RAWMANIA website instructions, with minor modifications and using aseptic technique. Chia seeds (3 teaspoons) were swollen in 75 mL sterile distilled water and psyllium (4 teaspoons) in 150 mL of sterile distilled water) for 15 min at laboratory temperature. The hydrated components were then blended with dates (4 pieces) and remaining ingredients (sunflower seed flour, 225 g; pumpkin seed flour, 100 g; sesame seeds, 3 spoons; Herbes de Provence, 1 spoon; dry yeast, 4 spoons; salt, 1 spoon) at 15,000 rpm for approximately 2 min.

The prepared blend was divided into four equal portions. One portion was inoculated with 1.0 mL of a *Lactobacillus acidophilus* suspension in physiological saline (equivalent to ~5.9 log CFU/g (LA blend)). A second portion received 1.0 mL of a *Fusarium langsethiae* spore suspension (~4.1 log CFU/g; FU). A third portion was inoculated with a 1:1 mixture of both suspensions (LAFU blend). Inocula were applied dropwise at multiple locations across the sample and the material was re-blended at 15,000 rpm for 2 min; however, spatial uniformity of microbial distribution was not verified. The remaining portion served as a negative control (NC). This entire procedure was repeated four times on separate days to obtain four independent replicate preparations for each experimental group (NC, LA, FU, LAFU), which were analyzed separately for microbiological parameters and mycotoxin content.

For drying, each portion was cut into 0.5 mm oval slices, arranged on a grid, and dried in a thermostat at 41.5 °C for 5 h to produce the MHT blend. The blend and MHT blend samples were then kept in the dark at ambient temperature for 24 h prior to microbiological analysis, and measurement of dry matter content and water activity. A subset of samples was subsequently freeze-dried at −110 °C for 16 h using the L4-110 Pro (Gregor Instruments s. r. o., Sázava, Czechia), ground to a powder in a ceramic mortar, and stored in plastic tubes at −80 °C until chemical analysis (mycotoxins, total phenolic content, and antioxidant activity).

### 2.5. Analysis of Mycotoxins

Sample preparation for LC–MS analysis was based on the procedure described by Pernica et al. [11] with minor modifications. Portions of 25.0 ± 0.1 g were combined with 100 mL of an acetonitrile/water mixture (84:16, *v*/*v*). The suspensions were shaken for 50 min and then centrifuged at 4000 rpm for 15 min. An 8 mL aliquot of the resulting supernatant was transferred into a glass tube and cleaned using a MycoSep^®^ 227 Trich+ column. From this, 4.0 mL of extract was evaporated to dryness in a rotary evaporator (IKA^®^ RV10 Digital, IKA, Staufen, Germany) and the residue was reconstituted in 1 mL of 10% methanol. The prepared solution was used directly for subsequent analysis. The *F. langsethiae* strain used in this study had previously been identified as a major producer of trichothecene mycotoxins (*Tri5* gene positive) [11]; therefore, the analysis focused on deoxynivalenol (DON), diacetoxyscripenol (DAS), neosolaniol (NEO), HT-2, and T-2 toxins. Determination was performed on a Waters Acquity UPLC system coupled to a XEVO TQ-S micro triple quadrupole mass spectrometer (Waters, Milford, MA, USA), following the approach of Martiník et al. [21]. Rapid polarity switching between positive (ESI^+^) and negative (ESI^−^) electrospray ionization modes was applied for mycotoxin identification and quantification. Data acquisition and processing were carried out using MassLynx™ (v4.2) and QuanLynx^®^ (v4.2) software (Waters, Manchester, UK). Chromatographic separation was achieved on a Waters Acquity UPLC^®^ BEH C18 column (2.1 × 100 mm, 1.7 μm; Waters, Wexford, UK) using a mobile phase composed of eluent A (1 mmol/L ammonium acetate + 0.5% acetic acid + 0.1% formic acid in water) and eluent B (0.5% acetic acid + 0.1% formic acid in methanol). The gradient program was: 0 min, 90% A; 3.0 min, 90% A; 10.0 min, 30% A; 10.1 min, 10% A; 12.0 min, 10% A; 13.1 min, 90% A; and 15.0 min, 90% A. The flow rate was maintained at 0.4 mL/min, injection volume was 10 μL using a flow-through needle mode, and the column temperature was set to 40 °C. The mass spectrometer was operated in ESI mode under the following parameters: capillary voltage, 1 kV; cone voltage, 30 V; source temperature, 120 °C; desolvation temperature, 450 °C. Argon was used as the collision gas and nitrogen as the nebulizing/desolvation gas, with a cone gas flow of 100 L/h and desolvation gas flow of 800 L/h. Analyses were performed in multiple reaction monitoring (MRM) modes, monitoring two transitions for each mycotoxin [21].

### 2.6. Moisture Content and Water Activity

The AquaLab TDL Water Activity Meter (Decagon Devices, Inc., Pullman, WA, USA) was used to measure water activity at 25 °C. Dry matter was determined by using a moisture analyzer Kern DLB 160-3A (KERN & SOHN GmbH, Balingen, Germany), at 105 °C, to a constant weight. The results were expressed in percentage of dry matter.

### 2.7. Total Phenolic Content and Antioxidant Activity

An aliquot of 2.0 g freeze-dried sample was extracted with 8.0 mL of 80% (*v*/*v*) methanol containing a drop of ethyl acetate. The mixture underwent sonication for 30 min, followed by centrifugation at 4000× *g* for 15 min (NF 400, Nüve, Ankara, Turkey), after which the supernatant was collected. Total phenolic content (TPC) was determined using the Folin–Ciocalteu method, which relies on the reduction of a phosphomolybdic–phosphotungstic acid complex by phenolic compounds, resulting in the formation of a blue-coloured complex. The reaction mixture was measured spectrophotometrically at 765 nm, following the procedure described in our previous work [22]. Gallic acid was employed for calibration, and results were expressed as gallic acid equivalents per gram of dry weight (mg GAE/g DW). Antioxidant capacity was assessed using the stable radical 2,2-diphenyl-1-picrylhydrazyl (DPPH) assay. Absorbance was measured at 517 nm, and the percentage of inhibition (*I*) was calculated according to the equation:

*I* [%] = ((*A*_0_ − *A*_1_)/*A*_0_)·100, (2)

where *A*_0_ represents the absorbance of the blank and *A*_1_ the absorbance of the sample. The reduction in DPPH absorbance, resulting from antioxidant compounds in the sample that donate either electrons or hydrogen atoms, was recorded. This reaction led to the decolourization of the purple DPPH radical solution, and the radical scavenging capacity was expressed as Trolox equivalent antioxidant capacity (mg Trolox/g DW) [22]. For each sample, two independent extracts were prepared, and each extract was analyzed in duplicate using spectrophotometry, resulting in a total of four measurements (N = 4).

### 2.8. Statistical Analysis

The type of material (blend vs. MHT blend) and experimental conditions (NC, LA, FU, and LAFU blends) were used as experimental factors influencing mycotoxin content. Data preprocessing included the Box–Cox transformation to normalize data and stabilize variance, ensuring compliance with the assumptions of normality and homogeneity of variances required for statistical methods such as analysis of variance (ANOVA). Normality was verified using the Shapiro–Wilk test, and homogeneity of variances was assessed using the Levene test. Mycotoxin measurements reported as below the analytical limit of detection (LOD) were treated as left-censored data. In accordance with established guidance for handling left-censored environmental and food contaminant datasets, these values were substituted with one-half of the LOD (LOD/2) to enable their inclusion in statistical analyses. This substitution method is widely recommended as a conservative approach that avoids overestimation (which may occur when substituting with the LOD) and underestimation (when substituting with zero), while preserving the distributional characteristics of low-level results for parametric analyses [23,24,25]. Principal component analysis (PCA) was then applied to the mycotoxin concentration dataset (DAS, NEO, HT-2, and T-2) from all experimental groups and replicates. The transformed data were further standardized to ensure comparability across variables measured on different scales. PCA was conducted on the covariance matrix, and the first two principal components, together explaining more than 97% of the total variance, were retained for interpretation. Scores (projections of samples in the new principal component space) and loading plots (showing the contribution and direction of individual mycotoxins to the principal components) were generated to visualize clustering patterns among samples and the relative influence of each variable. By reducing the dimensionality of the dataset, PCA preserved the majority of the variance, enabling clearer interpretation of the experimental results and revealing hidden structures within the data [26]. The scores of the dominant principal components, representing the main sources of variability in the dataset, were then used as dependent variables to evaluate the effects of the experimental factors. Scheffe’s post hoc tests were conducted to identify specific differences between groups. This approach effectively combines the data reduction capability of PCA with the statistical power of ANOVA, an established method for analyzing multivariate data by focusing on principal components that explain the majority of variance [27]. All analyses were evaluated at a significance level of 0.05 using MATLAB R2024b (The MathWorks, Inc., Natick, MA, USA).

## 3. Results and Discussion

### 3.1. The Microbial Load in Ingredients for Blend and MHT Blend Production

Microbiological analysis of the individual raw materials for the blend preparation revealed varying levels of contamination (Table S2). Pumpkin flour 1 showed a high burden, with *Aspergillus* spp., *Penicillium* spp., and the genus *Mucor*. *Bacillus* spp. and yeast were also detected. Consequently, this batch was discarded and replaced with pumpkin flour from other manufacturers (pumpkin flour 2 and 3). By contrast, pumpkin flours 2 and 3, sunflower flour, sesame, and dried yeast exhibited poor growth on MYP, DRBC, and DG18, with colony-forming units below the limit of detection (LOD). Chia seeds contained xerophilic fungi at 3.4 log CFU/g, and dates yielded *Aspergillus brasiliensis*. Psyllium harboured *Bacillus licheniformis*, *Cronobacter sakazakii*, *Penicillium* spp., and yeasts, while Herbs de Provence were contaminated with coliform bacteria (2.9 log CFU/g).

In the blend and MHT blend samples, TVC values ranged from 3.0 to 3.3 log CFU/g. Both matrices contained bacteria cells (*Bacillus licheniformis* and *Cronobacter sakazakii*), moulds (*Aspergillus* spp., *Penicillium* spp., and *Mucor* spp.), and yeast (Table 1), indicating carry-over from contaminated ingredients. TCBC values were 2.5–2.6 log CFU/g across all samples. TYMC values were 5.9 log CFU/g for FU and LAFU blends, increasing to 7.4–7.5 log CFU/g after minimal heat treatment. Drying at 41.5 °C for 5 h did not suppress mould growth, counts increased instead. We acknowledge that this result is unexpected, as drying typically reduces mould loads. We propose that in the early phase of drying, when moisture was still high and temperature increased gradually, transient permissive microenvironments allowed limited fungal proliferation before sufficient dehydration occurred, consistent with the findings of Hunaefi et al. [28]. Several *Fusarium* species tolerate—and in some cases grow at—moderately elevated temperatures (~30–40 °C) in a matrix- and a_w_-dependent manner [29], and high *a*_w_ is a key determinant of growth and toxin biosynthesis [30]. Thus, rather than 41.5 °C per se promoting growth, the combination of high *a*_w_ and slow warm-up likely supported residual activity during drying.

Indeed, temperatures of 40 °C for 10 h or 49 °C for 4.5 h were found to be optimal for the preparation of two white corn flour starters consisting of ten fungi species [28]. In our research, *L. acidophilus* was detected in LA and LAFU blends at the level of 4.8 log CFU/g, both before and after the drying process. The TYMC values for FU and LAFU blends were the same, indicating that the presence of *L. acidophilus* did not alter the growth of *F. langsethiae* (Table 1). This is contrary to the findings of Salah-Abbès et al., who found that both viable cells of *L. plantarum* and cell-free supernatant inhibited the mycelial growth of *F. graminearum* on the surface of maze agar by 79 and 42%, respectively [31].

### 3.2. Moisture Content and Water Activity of Blends

Water activity in the blends ranged from 0.966 to 0.971 and was significantly reduced to 0.934–0.939 in the NC, LA, and FU MHT blend samples (*p* < 0.05). Dry matter content was 75.4–77.0 and 76.3–78.3% in blend and MHT blend samples, respectively (Table 2). Although drying at 41.5 °C for 5 h lowered a_w_, the accompanying increase in dry matter content was not significant (*p* > 0.05).

### 3.3. Total Phenolic Content and Antioxidant Activity of Blends

The total phenolic content ranged from 4.11 to 6.01 mg GAE/g DW in the blends and increased to 5.57–8.59 mg GAE/g DW in MHT blends (*p* < 0.05). A more detailed view showed that negative control exhibited similar TPC values before and after drying, whereas air-drying at 42.5 °C for 5 h significantly increased TPC from 4.11 to 7.64 (*p* < 0.001), 6.01 to 8.59 (*p* < 0.001), and 5.48 to 6.89 mg GAE/g DW (*p* < 0.05) in LA, FU, and LAFU samples, respectively (Table 2). While the NC sample showed no changes, the higher TPC observed in the MHT samples is assumed to be associated with the presence of lactobacilli and moulds; however, this interpretation remains speculative and warrants further investigation. An increase in phenolics during solid-state fermentation has been well documented [32]. In that report, solid-state fermentation of corn bran samples by *L. reuteri* and *L. plantarum* for 24 h resulted in a three- to fourfold rise in total phenolic content. However, this does not explain the TPC increase observed in MHT blends inoculated with *F. langsethiae*. Some *Fusarium* species are known to produce phenols and flavonoids as secondary metabolites [33] and can metabolize plant-derived phenolic acids in ways that may influence antifungal activity [34]. These potential interactions require further investigation to clarify their role in the observed TPC increase. Antioxidant capacity was higher in the MHT blends than in the blends before drying; however, this increase was not statistically significant (*p* > 0.05).

### 3.4. Mycotoxin Content in Blend and HMT Blend

Since the inoculation of fungal spores and lactobacilli cells took place in a semi-solid medium, the homogenization of the samples appeared to be the most problematic part of the experiment. Most of the published experiments investigating the effects of *Lactobacillus* strains on mycotoxin production were performed in a liquid laboratory medium that is optimal for them (i.e., MRS) [14,15]. This experimental design facilitates the acquisition of precise results, however its application to semi-solid or solid food matrices is challenging. It should be noted that the semi-solid matrix used here was not a standardized food model, and microbial distribution may therefore have been uneven. Such non-uniformity can create localized “hot spots” of growth and mycotoxin formation, and may inflate between-replicates variability. Accordingly, CFU and mycotoxin values should be interpreted as composite read-outs of a heterogenous system rather than fully homogenized averages. The formation of mycotoxins as well as the growth of lactobacilli may not take place throughout the mass of the samples. The penetration of mycotoxin from mould’s cells to the entire mass of the sample is limited by many biological (fungal strain and its growth, mycotoxin molecule size, and its hydrophobicity), food-related (composition, texture, water activity, pH) factors [35]. We recognize that pH and key organic acid (lactic and acetic) were not measured in this study. Given their potential role in modulating *Fusarium* growth and toxin production, the absence of these data represents a limitation and should be addressed in follow-up studies to strengthen the mechanistic interpretation. In our research with pumpkin–sunflower seed flour blends, the DAS content was below the limit of detection in two individual experiments for both FU and LAFU samples, while NEO was below the limit in one experiment. Additionally, HT-2 and T-2 toxins were not detected in LAFU samples in one individual experiment (Table 3). After production of MHT blends, mycotoxins were successfully quantified in all the samples, with the exception of FU (two individual experiments) and LAFU (one individual experiment). Deoxynivalenol was below LOD in all the samples. This fact does not necessarily indicate its complete absence, as some *Fusarium* species have been reported to produce masked forms of DON [36]. These derivates, such as acetylated or glycosylated conjugates, may evade detection by conventional analytical methods. Since the method applied in this study was not designed to identify masked mycotoxins, their potential presence cannot be excluded.

**Figure 1:**
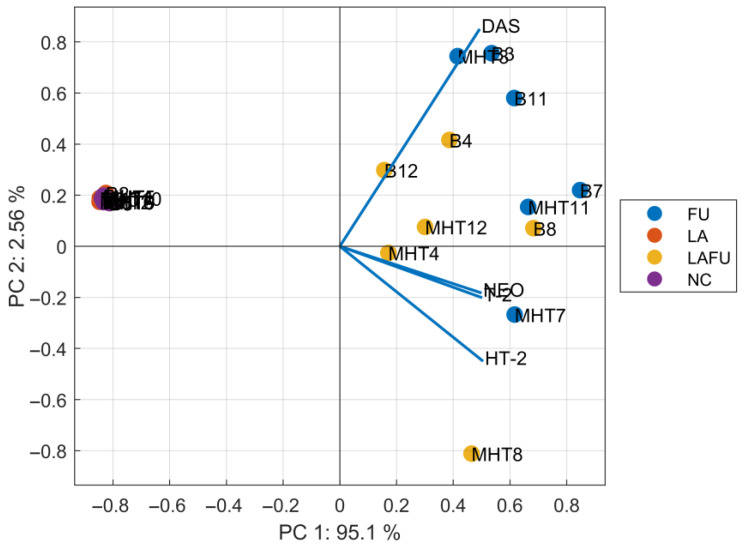
Biplot illustrating the principal components analysis between mycotoxin content and experimental conditions for each replicate. Blend (B); minimally heat-treated blend (MHT); the number indicates individual measurements; Negative control (NC); blend inoculated with *Lactobacillus acidophilus* (LA); blend inoculated with *Fusarium langsethiae* (FU); blend inoculated with *L. acidophilus* and *F. langsethiae* (LAFU). NC and LA groups, in which most mycotoxin concentrations were at or below the LOD, clustered tightly together and apart from all *Fusarium*-inoculated samples (FU and LAFU), indicating that low- or non-detectable toxin profiles were consistently captured in the PCA. MHT minimally-heat treated; DAS, diacetoxyscripenol; NEO, neosolaniol.

Values below the LOD were processed according to the procedure described in Section 2.8, enabling their inclusion in the statistical analyses. PCA was applied to the normalized dataset to identify latent structure and reduce dimensionality. The resulting biplot (Figure 1) displays both sample scores and variable loadings, providing insights into clustering patterns, relationships among experimental conditions, and the contributions of individual mycotoxins to the principal components. This approach allowed clearer interpretation of underlying trends and, highlighted key differences and associations driven by the experimental factors. The PCA biplot illustrates the relationships between experimental conditions and *Fusarium*-derived metabolites (e.g., T-2, HT-2, NEO, and DAS) while also showing the clustering of experimental samples. The first principal component (PC1) explained 95.1% of the total.

Variance represents the primary source of variability, largely driven by the production of *Fusarium* metabolites under conditions involving *Fusarium* (FU) or *Lactobacillus acidophilus* combined with *Fusarium* (LAFU). The second component (PC2), explaining 2.56% of the variance, captures smaller variations across conditions. Samples from the NC (negative control) and LA (without *Fusarium*) conditions cluster tightly in the lower left quadrant of the biplot, reflecting the absence of *Fusarium* metabolites. In contrast, FU samples were distinctly separated along PC1, aligning with vectors representing *Fusarium* metabolites (e.g., T-2, HT-2, NEO, DAS) indicating high production of these compounds under *Fusarium*-dominated conditions. LAFU samples were positioned between the FU and LA clusters, consistent with partial suppression of mycotoxin production when co-inoculated with *Lactobacillus acidophilus*. Loading vector analysis indicated that PC1 was primarily driven by HT-2, NEO, and DAS, with T-2 contributing to a lesser extent, while PC2 was strongly influenced by T-2 and negatively by NEO. The intermediate position of LAFU reflected marked reductions in HT-2 and NEO relative to FU, whereas T-2 levels remained higher than in LA but lower than in FU. This toxin-specific profile suggests that *L. acidophilus* modulates *Fusarium* metabolism in a non-uniform manner, exerting greater effects on certain trichothecenes (e.g., HT-2) than others (e.g., T-2). Several approaches or mechanisms have been proposed to explain how lactic acid bacteria influence fungal growth and mycotoxin production. Arena et al. [37] stated that the protonated form of lactic acid disrupts the pH gradients between the acidic exterior and the alkaline cytosol, leading to dissipation of the membrane potential and ultimately cell death. However, acidic pH has been shown to promote DON production in *F. graminearum* [38], so suppression by lactic acid or acetic acid alone is not expected. Notably, citric acid can impair trichothecene biosynthesis even as fungal growth persists, indicating that growth and toxin production can be decoupled in some contexts [39]. This effect has not been demonstrated for lactic or acetic acid, and may be species- and matrix-dependent. Thermal transformation of trichothecenes is typically reported only under baking- or extrusion-like processing at >140–200 °C [40]. By contrast, recent evidence indicates that structural changes can also arise under moderate conditions via biology: several *Bacillus* strains degraded DON at 25–42 °C and generate derivative/isomeric products (e.g., M-DON, norDON E, 9-hydroxymethyl DON lactone) [41] and, at 20–25 °C with elevated water activity, conjugated forms such as HT-2-glucoside and DON-3-glucoside cam increase [42]. Although viable *L. acidophilus* failed to inhibit *F. langsethiae* growth in our experiment, mycotoxin concentration nevertheless declined. Combined with prior in vitro findings of equivalent DON removal by viable and heat-inactivated cells, with no detectable DON metabolites [16], this supports passive adsorption to cell-wall peptidoglycan and polysaccharides as the principal mechanism [14,31]. Given above, the 41.5 °C drying step is far below temperatures at which thermal chemistry is expected. Any post-drying variations are therefore plausibly attributable to residual fungal biosynthesis in high a_w_ microenvironments than to heat-induced reactions. Future work should explicitly monitor modified forms and consider microbially mediated transformation under moderate conditions. The PCA separation remained robust despite the inclusion of values at or below the limit of detection (LOD), substituted with LOD/2, which predominantly reflected biologically favourable conditions with suppressed mycotoxin production and did not alter the principal component structure or the clear separation between high-toxin (FU) and low-toxin (NC, LA, selected LAFU) clusters.

To corroborate the PCA, a two-way ANOVA was performed on PC1 scores (capturing > 95% of the variance), providing a compact summary while avoiding overfitting. Material type (blend vs. MHT blend) and experimental conditions (NC, LA, FU, LAFU) were tested. Only the experimental condition was significant (*p* < 0.05), whereas material type and the interaction were not (Table S3). Sheaffe’s post hoc tests indicated that all *Fusarium*-involving groups differed from the others. And FU also differed from LAFU (*p* < 0.05; Table S4), consistent with modulation by *L. acidophilus.* The tested blend, commercially available with preparation instructions on a vegan website, represents a realistic minimally processed product. Its relatively high a_w_ (0.93–0.94) and moderate dry matter (76–78%) can support *Fusarium* growth and mycotoxin production, raising a potential food-safety concern. Because the semi-solid matrix is viscous, inoculum distribution was likely non-uniform, which can increase variance and bias absolute levels upward and downward. The principal trends remained detectable in PCA/ANOVA, suggesting robustness to this heterogeneity. Nevertheless, additional experiments would increase statistical power, particularly where many values lie at or below the LOD. The addition of lactic acid bacteria may reduce the mycotoxin burden in such raw-style mixtures. However, the effects on bioavailability and overall risk assessment require more in-depth analysis.

## Figures and Tables

**Table 1:** Microbial counts in blend and minimally heat-treated (MHT) blend.

Sample		Viable Count (log CFU/g)				
		TMC	BCC	TCBC	LBC	TYMC
NC	blend	3.3	<2.0	2.5	<2.0	<2.0
	MHT blend	3.1	<2.0	2.6	<2.0	<2.0
LA	blend	3.1	<2.0	2.6	4.8	<2.0
	MHT blend	3.2	<2.0	2.5	4.8	<2.0
FU	blend	3.1	<2.0	2.6	<2.0	5.9
	MHT blend	3.3	<2.0	2.6	<2.0	7.5
LAFU	blend	3.1	<2.0	2.6	4.8	5.9
	MHT blend	3.0	<2.0	2.6	4.8	7.4

Negative control (NC); sample inoculated with *Lactobacillus acidophilus* (LA); sample inoculated with *Fusarium langsethiae* (FU); sample inoculated with mixture of *Lactobacillus acidophilus* and *Fusarium langsethiae* (LAFU); Total viable count (TMC); Presumptive *Bacillus cereus* count (BCC); Total coliform bacteria count (TCBC); Total yeasts and moulds count (TYMC); Lactic acid bacteria count (LBC).

**Table 2:** Water activity, moisture content, total phenolic content (TPC), and antioxidant capacity (AC) in the blend and the minimally heat-treated (MHT) blend.

Sample		Water Activity	Dry Matter (%)	TPC (mg GAE/g DW)	AC (mg Trolox/g DW)
NC	blend	0.967 ± 0.009 ^a^	77.0 ± 1.9 ^a^	5.38 ± 0.58 ^d^	14.07 ± 3.62 ^a^
	MHT blend	0.934 ± 0.008 ^b^	78.3 ± 0.8 ^a^	5.57 ± 1.40 ^d^	13.65 ± 1.82 ^a^
LA	blend	0.970 ± 0.003 ^a^	75.4 ± 1.4 ^a^	4.11 ± 0.56 ^e^	13.23 ± 0.93 ^a^
	MHT blend	0.936 ± 0.006 ^b^	76.3 ± 0.6 ^a^	7.64 ± 0.68 ^cb^	16.65 ± 1.28 ^ab^
FU	blend	0.971 ± 0.012 ^a^	76.5 ± 0.7 ^a^	6.01 ± 0.28 ^c^	16.07 ± 3.38 ^a^
	MHT blend	0.934 ± 0.008 ^b^	77.0 ± 0.3 ^a^	8.59 ± 1.06 ^a^	19.02 ± 2.53 ^a^
LAFU	blend	0.966 ± 0.012 ^a^	75.6 ± 2.3 ^a^	5.48 ± 0.45 ^d^	13.93 ± 1.16 ^a^
	MHT blend	0.939 ± 0.017 ^a^	76.8 ± 0.1 ^a^	6.89 ± 0.70 ^bc^	15.43 ± 2.41 ^ab^

Negative control (NC); blend inoculated with *Lactobacillus acidophilus* (LA); blend inoculated with *Fusarium langsethiae* (FU); blend inoculated with *L. acidophilus* and *F. langsethiae* (LAFU); different superscript letters indicate statistically significant difference in a column (*p* < 0.05); mean ± standard deviation (*N* = 4).

**Table 3:** Mycotoxin content (µg/kg) in individual experiments.

	Sample	DAS	NEO	HT-2	T-2
blend 1	FU	<LOD	5.2	1.4	2.3
	LAFU	<LOD	3.7	1.1	1.3
blend 2	FU	<LOD	<LOD	1.4	0.2
	LAFU	<LOD	<LOD	<LOD	<LOD
blend 3	FU	0.17	35.6	17.1	5.7
	LAFU	0.08	19.1	8.4	2.4
MHT blend 1	FU	0.06	12.7	12.5	3.2
	LAFU	0.03	9.9	9.7	3.1
MHT blend 2	FU	<LOD	3.0	1.2	1.0
	LAFU	<LOD	2.5	0.7	0.9
MHT blend 3	FU	<LOD	1.0	11.3	30.3
	LAFU	0.05	0.5	1.8	4.2
LOD		0.01	0.04	0.09	0.06

MHT, minimally heat-treated; LA, blends inoculated with *L. acidophilus*; FU, blends inoculated with *F. langsethiae*; LAFU, inoculation with the mixture of *L. acidophilus* and *F. langsethiae*; DAS, diacetoxyscripenol; NEO, neosolaniol; LOD, limit of detection; DON < LOD in all samples.

## Data Availability

The data used in this manuscript are available upon request from the author.

## Supplementary Materials

The following supporting information can be downloaded at: https://www.mdpi.com/article/10.3390/foods14173077/s1, Table S1: Ingredients for minimally heat-processed bread production; Table S2: Microbial quality of the raw materials used to make blend; Table S3: Results of the two-way ANOVA performed on the principal scores of the first principal component (PC1); Table S4: Pairwise comparisons of experimental groups based on the principal scores of the first principal component (PC1).
