# Supplementary material for: Evaluating the Impact of Lactobacillus acidophilus on Fusarium Mycotoxins in Raw Vegan Pumpkin–Sunflower Seed Flour Blends
Source: Foods. 2025 Sep 1;14(17):3077. doi: 10.3390/foods14173077 (PMC12427764; doi:10.3390/foods14173077)
Supplement: Supplementary file 1 [file foods-14-03077-s001.zip › foods-3748121-supplementary.pdf]

Evaluating the impact of *Lactobacillus acidophilus* on *Fusarium* mycotoxins in raw vegan pumpkin-sunflower seed flour blends.

Iveta Brožková[1], Marek Pernica[2], Sylvie Běláková[2] Markéta Vydržalová[1], Petra Moťková[1], Ivana Stará[1], Lenka Husáková[3], Libor Červenka[3]

Table S1. Ingredients for minimally heat-processed bread production.

product	weight (g)	manufacturer	country of origin
seedless dates	250	Provita	Czech Republic
pumpkin seed flour 1	250	Ingredience	Czech Republic
pumpkin seed flour 2	300	Zdraví z přírody	Czech Republic
pumpkin seed flour 3	500	Natural	Czech Republic
chia seeds	500	Provita	Czech Republic
herbs de Provence	25	Sonnentor	Czech Republic
psyllium	100	Valdemar Grešík – Natura	Czech Republic
hulled sesame	100	Country life	Czech Republic
sunflower seed flour	300	Zdraví z přírody	Czech Republic
fine sea salt	1000	Country life	Czech Republic

Table S2. Microbial quality of the raw ingredients for the blend.

ingredient	viable count (log CFU/g)				
	**TMC**	**BCC**	**TCBC**	**TYMC**	**OXC**
pumpkin seed flour 1	< 3.0	4.0	< 1.0	3.0	3.2
pumpkin seed flour 2	< 3.0	< 3.0	< 1.0	< 2.0	< 2.0
pumpkin seed flour 3	< 3.0	< 3.0	< 1.0	< 2.0	< 2.0
sunflower seed flour	< 3.0	< 3.0	< 1.0	< 2.0	< 2.0
sesame	< 3.0	< 3.0	< 1.0	< 2.0	< 2.0
dry yeast	< 3.0	< 3.0	< 1.0	< 2.0	< 2.0
chia seeds	< 3.7	< 3.7	< 0.7	< 2.7	3.4
dates	< 3.0	< 3.0	< 1.0	< 2.0	< 2.0
psyllium	overgrown	< 3.7	3.5	< 2.7	< 2.7
herbs de Provence	< 3.7	< 3.7	2.9	< 2.7	< 2.7

Total microbial count (TMC); Presumptive *Bacillus cereus* coount (BCC); Total coliform bacteria count (TCBC); Total yeasts and moulds count (TYMC); Osmophilic yeasts and xerophilic moulds count (OXC)

Table S3. Results of the two-way ANOVA performed on the principal scores of the first principal component (PC1).

**source**	**sum sq.**	**df**	**mean sq.**	**F**	**prob > F**
material	0.115	1	0.115	2.94	0.185
group	85.1684	3	28.3895	725.92	0.0001
material: group	0.1173	3	0.0391	0.3	0.8257
error	2.0932	16	0.1308		
total	87.4939	23			

Material (blend and MHT bread), group (NC, LA, FU, and LAFU blends), and their interaction (material × group) on the dominant source of variability (captured by PC1). NC, negative control; LA, blends inoculated with *L. acidophilus*, FU, blends inoculated with *F. langsethiae*; LAFU, inoculation with the mixture of *L. acidophilus* and *F. langsethiae*

Table S4. Pairwise comparisons of experimental groups based on the principal scores of the first principal component (PC1).

group1	group2	CI (95%)	mean diff.	*p* value
FU	LA	3.507–4.702	4.104	4.869×10^{-12}
FU	LAFU	0.150–1.345	0.747	0.012
FU	NC	3.507–4.702	4.104	4.869×10^{-12}
LA	LAFU	-3.954– -2.759	-3.357	1.406×10^{-10}
LA	NC	-0.597–0.597	8.88×10^{-16}	1.0
LAFU	NC	2.759–3.954	3.357	1.406×10^{-10}

NC, negative control; LA, blends inoculated with *L. acidophilus*, FU, blends inoculated with *F. langsethiae*; LAFU, inoculation with the mixture of *L. acidophilus* and *F. langsethiae;* CI, 95% confidence interval;
